# A biomarker discovery framework for childhood anxiety

**DOI:** 10.3389/fpsyt.2023.1158569

**Published:** 2023-07-17

**Authors:** William J. Bosl, Michelle Bosquet Enlow, Eric F. Lock, Charles A. Nelson

**Affiliations:** ^1^Center for AI & Medicine, University of San Francisco, San Francisco, CA, United States; ^2^Computational Health Informatics Program, Boston Children’s Hospital, Boston, MA, United States; ^3^Department of Pediatrics, Harvard Medical School, Boston, MA, United States; ^4^Department of Psychiatry and Behavioral Sciences, Boston Children’s Hospital, Boston, MA, United States; ^5^Department of Psychiatry, Harvard Medical School, Boston, MA, United States; ^6^Division of Biostatistics, School of Public Health, University of Minnesota, Minneapolis, MN, United States; ^7^Laboratories of Cognitive Neuroscience, Division of Developmental Medicine, Boston Children’s Hospital, Boston, MA, United States; ^8^Harvard Graduate School of Education, Cambridge, MA, United States

**Keywords:** childhood anxiety, externalizing disorders, biomarkers, EEG, nonlinear analysis, computational psychiatry, tensor analysis

## Abstract

**Introduction:**

Anxiety is the most common manifestation of psychopathology in youth, negatively affecting academic, social, and adaptive functioning and increasing risk for mental health problems into adulthood. Anxiety disorders are diagnosed only after clinical symptoms emerge, potentially missing opportunities to intervene during critical early prodromal periods. In this study, we used a new empirical approach to extracting nonlinear features of the electroencephalogram (EEG), with the goal of discovering differences in brain electrodynamics that distinguish children with anxiety disorders from healthy children. Additionally, we examined whether this approach could distinguish children with externalizing disorders from healthy children and children with anxiety.

**Methods:**

We used a novel supervised tensor factorization method to extract latent factors from repeated multifrequency nonlinear EEG measures in a longitudinal sample of children assessed in infancy and at ages 3, 5, and 7 years of age. We first examined the validity of this method by showing that calendar age is highly correlated with latent EEG complexity factors (*r* = 0.77). We then computed latent factors separately for distinguishing children with anxiety disorders from healthy controls using a 5-fold cross validation scheme and similarly for distinguishing children with externalizing disorders from healthy controls.

**Results:**

We found that latent factors derived from EEG recordings at age 7 years were required to distinguish children with an anxiety disorder from healthy controls; recordings from infancy, 3 years, or 5 years alone were insufficient. However, recordings from two (5, 7 years) or three (3, 5, 7 years) recordings gave much better results than 7 year recordings alone. Externalizing disorders could be detected using 3- and 5 years EEG data, also giving better results with two or three recordings than any single snapshot. Further, sex assigned at birth was an important covariate that improved accuracy for both disorder groups, and birthweight as a covariate modestly improved accuracy for externalizing disorders. Recordings from infant EEG did not contribute to the classification accuracy for either anxiety or externalizing disorders.

**Conclusion:**

This study suggests that latent factors extracted from EEG recordings in childhood are promising candidate biomarkers for anxiety and for externalizing disorders if chosen at appropriate ages.

## 1. Introduction

Anxiety is the most common manifestation of psychopathology in youth, negatively affecting academic, social, and adaptive functioning and increasing risk for mental health problems into adulthood (1–4). Vulnerability may be established in early life: by 2 to 5 years of age, 10%–20% of children meet criteria for at least one anxiety disorder, including generalized anxiety disorder, social anxiety disorder, and separation anxiety disorder (5, 6). In addition, middle childhood may be a core risk period for the emergence or exacerbation of anxiety, given the transition to formal schooling and consequent dramatic changes in contextual, cognitive, and social demands (7, 8). Existing methodology can effectively diagnose anxiety disorders only after emergence of clinical symptoms, resulting in missed opportunities to intervene during critical early prodromal periods. Accordingly, there is a need for prospective studies beginning in infancy that elucidate the early precursors of anxiety to inform the development of risk identification tools, allowing for preventative intervention to be applied prior to the emergence of clinical symptoms.

Neurodevelopmental models of anxiety posit that vulnerability may arise from aberrant development of neural networks that mediate typical anxiety-related behaviors (9–13). These networks are hypothesized to be established during brief periods of heightened plasticity early in life (14). Molecular signals and neural maturation initiate the periods of plasticity when the organism becomes highly receptive to the environment and when relevant neurocognitive systems acquire their individual phenotypic characteristics (14, 15). The developmental timing of these processes in the human are poorly understood, however, given a paucity of studies using direct measures of brain activity and linking early brain development with later behavioral symptoms of anxiety. Moreover, although different anxiety phenotypes demonstrate distinctive features, they also share core characteristics, and comorbidity among anxiety disorders is common (16–19). Thus, there is a need to identify transdiagnostic markers that reflect underlying neural processes common across anxiety disorders/phenotypes (20).

Establishing measurable indicators of anxiety vulnerability in early childhood may provide much needed risk biomarkers that enable early, preventive intervention. Even if the earliest biomarkers for anxiety risk do not yield a high degree of specificity, discovery of neurophysiological markers that indicate higher likelihood of later psychopathology holds great promise for identifying potentially at-risk children. Such identification tools would allow monitoring procedures to be implemented and, as indicated, strategies that target and re-direct underlying neurodevelopment in more optimal trajectories could be applied, thereby reducing the onset of clinical symptoms and associated adverse outcomes. Additionally, identification of predictive or concurrent neural biomarkers of anxiety may lead to discoveries of novel causal pathways, which could inform the development of more targeted, effective treatment and prevention efforts (21). Repeated assessments across early childhood are important to determine the developmental time-course of the neural processes involved in the pathophysiology of anxiety; the earliest age at which neural biomarkers are reliably predictive of anxiety risk; and the relative robustness of trajectories of neural functioning measures relative to measures at a given age in predicting child anxiety outcomes. Such information will allow for more precise identification methods and treatments to correct atypical processes and their downstream behavioral manifestations.

To date, neural signatures that are associated with psychopathology with high sensitivity and specificity remain elusive. Functional magnetic resonance imaging (fMRI) can predict individual differences in anxiety during middle childhood (22) and treatment responsivity in adults (23). However, fMRI is costly and complex, making it unlikely to be scalable for use as a general screening tool. A promising alternative approach is analysis of neural activity patterns measured via electroencephalogram (EEG) recordings. Efforts thus far to utilize EEG data for predicting mental health status and mental health trajectories have primarily applied traditional spectral power analysis. For example, frontal and parietal alpha resting-state EEG asymmetry have been linked to anxiety and related risk markers (e.g., threat biases, behavioral inhibition) (24–26). However, the predictive power of spectral power is limited.

A review of both event-related potentials (ERPs) and evoked Potentials (EPs) has demonstrated that both approaches have some ability to differentiate children with high anxiety levels from controls (27). Biomarkers for anxiety disorders have been extracted from EEGs using ERPs to discover differences between participants (21, 28–30). For example, the error-corrected negativity (ERN) ERP has been found to be increased in anxious youth and to predict increased risk for anxiety across development (31). A systematic review of EEG research found that the ERN is a promising biomarker of clinical anxiety (19). Additionally, ERP studies based on startle reactivity have differentiated different internalizing phenotypes from each other and controls, also supporting the potential of this measure as an anxiety biomarker (32). EEG measures extracting from both resting state and task based recordings, including power spectra, Higuchi fractal dimension (a nonlinear measure), and correlation indices (pairwise measures), all contributed to a machine learning biomarker for major depressive disorder (33), suggesting that linear and nonlinear measures, resting state or task-based, may be useful for psychiatric biomarkers.

A theoretical foundation for using nonlinear analysis of brain function using EEG is based on a conceptualization of the brain as a dynamical system and the growing understanding of the information processing capacity of dynamical systems (34). Nonlinear measures are an attempt to reverse engineer quantitative measures of brain dynamics from time series measurements, using a well-known process called time series embedding. See Chapter 9 of (35) for a discussion of time series embedding in the context of neuroscience. Nonlinear analysis of EEG time series is a promising approach to functional brain analysis and biomarker discovery (36–38) and has demonstrated potential for very early prediction of emerging autism spectrum disorder (36, 39, 40), ADHD (41, 42), detection and monitoring of epilepsy (43–45), and measuring sleep disorders (46), among many others. However, the large number of nonlinear measures that can be computed (47), including different approaches to frequency or scale decomposition (48), and multiple scalp sensor arrangements create very large multidimensional sets of quantitative values that pose analytical challenges.

One of the goals of our project was to develop computational methods that would enable latent features to be extracted from multiple EEG-derived measures to enable the relative importance of the growing number of EEG features to be explored. Although we do not address the full range of quantitative measures that may be derived from EEGs, such as those described above, we use a relatively large number of nonlinear measures (plus traditional spectral power) as inputs to a tensor factorization algorithm to extract a much smaller number of latent factors. We hope that other researchers may find supervised tensor factorization useful for evaluating the contribution of additional EEG or physiological measures to latent biomarker discovery.

We have previously demonstrated that nonlinear EEG analysis applied to typically developing infants and infants at high risk for autism spectrum disorder based on family history was able to reliably predict which infants would later develop autism (36, 49–51). Anxiety disorders have been described as dynamical disorders (52), suggesting that dynamical biomarkers for anxiety may be discovered. Here, we used an empirical approach to nonlinear EEG analysis with the goal of discovering differences in brain electrodynamics that distinguish children with anxiety disorders from children who do not have this condition. Importantly, EEG is a widely available, low-cost, easy-to-administer, time-efficient method that, as technology advances (e.g., advanced ambulatory methods), could be incorporated into routine clinical practice, transforming opportunities for wide screening for risk and preventative intervention (30, 53).

The overall objective of the current study was to compute a set of nonlinear measures across traditional frequency bands from EEG data assessed repeatedly in early life, then use a supervised tensor factorization approach to extract latent features from the nonlinear measures to test whether these features are associated with anxiety diagnosis by middle childhood. To our knowledge, this is the first use of supervised tensor factorization for extracting latent nonlinear features from EEG signals. We used the algorithm SupCP – “Supervised Canonical Polyadic” factorization – in this research (54). To demonstrate the utility of SupCP to extract latent features, we first applied this method to predict the calendar age of participants from nonlinear EEG features. We then compared group differences in the latent features from participants diagnosed with either an anxiety disorder or an externalizing disorder versus participants with no psychiatric history (healthy controls) to identify neural activity signatures predictive of anxiety diagnoses. We included an externalizing disorder group to test the specificity of any findings to anxiety versus general psychopathology. We conducted these analyses in a community-based longitudinal cohort designed to examine how neural biomarkers and other risk factors in early life contribute to childhood anxiety risk. The dataset includes repeated EEG assessments, conducted in infancy (5-, 7-, or 12 months of age), 3 years, 5 years, and 7 years of age, and detailed clinical assessments of child anxiety and other psychopathology diagnostic history at age 5 years. By considering repeated assessments from infancy through middle childhood, we can determine if there are sensitive risk periods and clarify if two or more EEG recordings more robustly predict anxiety than assessment at a single time point. Importantly, we present for the first time a novel latent feature extraction methodology based on supervised tensor factorization that allows clinical data to inform the extracted latent EEG features. The latent features are interpretable, making this methodology a potentially valuable discovery tool. This approach to integrated EEG and patient multimodal data analysis may be a useful addition to computational psychiatry (55, 56) and mental health informatics (57) research toolkits.

## 2. Methods

### 2.1. Participants

The current analyses use data from an ongoing longitudinal cohort study on the development of emotion processing during the first years of life. Participants (hereafter referred to as “children”) were recruited from a registry of local births comprising families who had indicated willingness to participate in developmental research. Exclusion criteria included known prenatal or perinatal complications, pre-term or post-term birth (±3 weeks from due date), developmental delay, uncorrected vision difficulties, and neurological disorder or trauma. After enrollment, families were no longer followed and their data were excluded from analyses if the child was diagnosed with an autism spectrum disorder or if a genetic or other condition known to influence neurodevelopment was discovered (e.g., hydrocephalus; absence seizures; brain tumor; maternal use of anticonvulsants, antipsychotics, opioids in pregnancy).

Families were enrolled in the study when the children were (randomly) 5, 7, or 12 months old (infancy assessment), with a subsample followed when the children were 3, 5, and 7 years of age. The current analyses began after completion of all 3 year assessments, with 5- and 7 years assessments ongoing. By design, approximately half of the cohort was randomly assigned to be administered a protocol to collect EEG and ERP data at each assessment, and the other half of the sample to be administered a protocol to collect functional near-infrared spectroscopy (fNIRS) data at each assessment. At age 5 years, all children’s mothers were invited to complete a semi-structured clinical interview to assess child lifetime psychiatric diagnoses. There were no differences between children who completed the EEG/ERP versus fNIRS protocol on child sex assigned at birth, race/ethnicity, or lifetime anxiety diagnoses, *p*s > 0.35. Children were eligible for inclusion in the current analyses if they provided EEG data at infancy, 3 years, and/or 5 years and clinical diagnostic data at 5 years. This resulted in an analytic sample size of 150 children, although the number of children measured at each developmental time point (infancy, 3, 5, 7 years) is less than this total.

### 2.2. Procedures

Children in the current analyses were invited in infancy and at ages 3, 5, and 7 years to participate in a laboratory visit that included measurements of baseline EEG. At age 5 years, the child’s mother was administered a semi-structured clinical interview that assessed the child’s lifetime clinical psychiatric diagnostic history. Sociodemographic data were obtained at study enrollment and at subsequent assessments as relevant via online questionnaires completed by the child’s parent (primarily the child’s mother). Study procedures were approved by the Institutional Review Board of Boston Children’s Hospital, and parents provided written informed consent prior to the initiation of any study activities; at age 7 years, children provided assent.

#### 2.2.1. EEG recording, processing, and analysis

**Figure 1:**
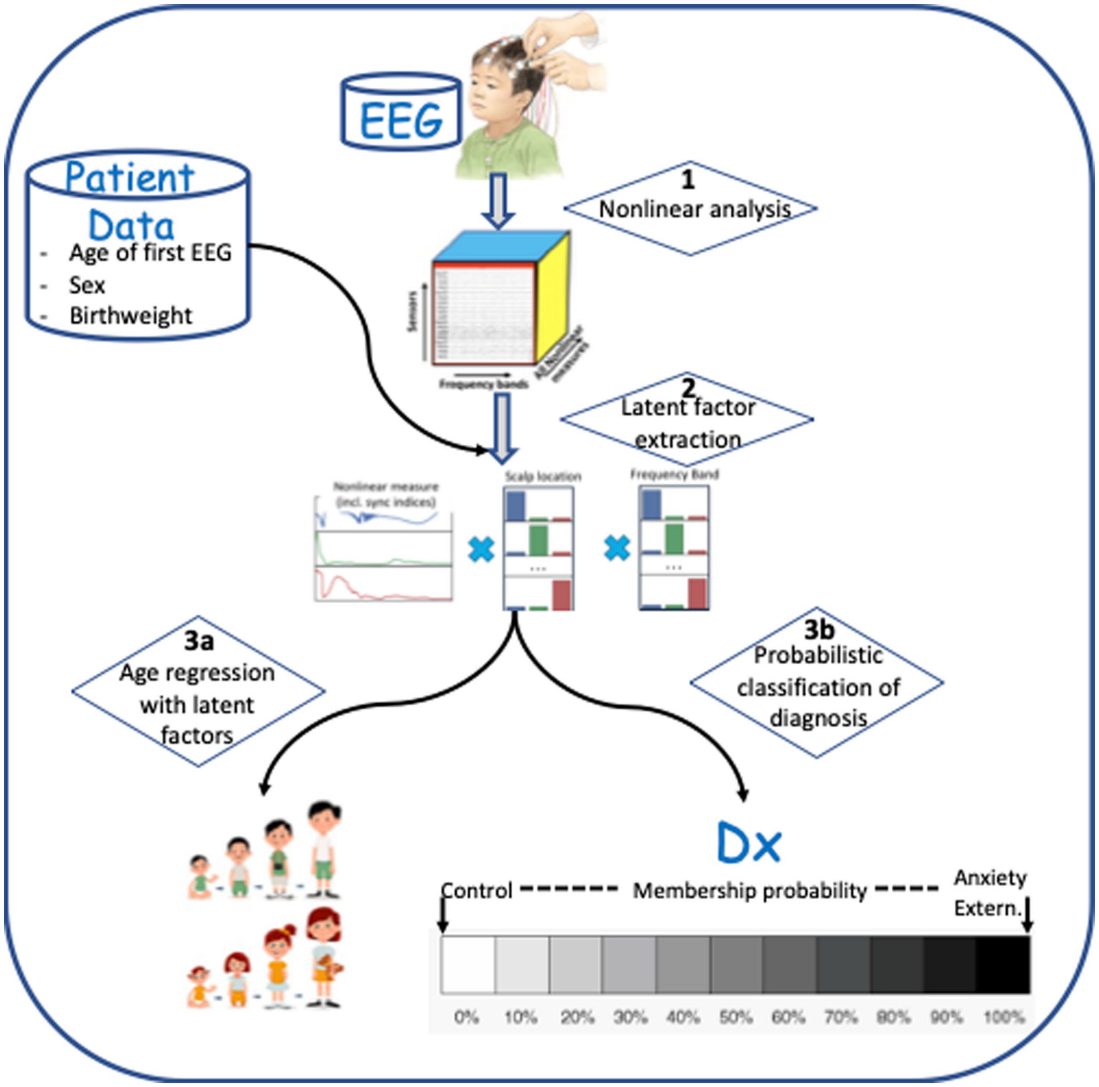
Data processing steps involved in analysis. (1) Multi-frequency decomposition of the signal and computation of nonlinear measures. (2) Latent feature extraction using Supervised Canonical Polyadic (SupCP) algorithm, including EEG measures and covariate data. (3a) Predicting age using regression with latent features. (3b) Classification of anxiety disorder group versus healthy control group, externalizing disorder group versus healthy control group, or anxiety disorder group versus externalizing disorder group.

Continuous scalp EEG was recorded from a 128-channel HydroCel Geodesic Sensor Net (HCGSN; Electrical Geodesic Inc.), referenced to the vertex electrode (Cz), and sampled at 500 Hz. Impedances were kept at or below 100 kiloohms. Thirty-second awake resting state EEG segments were selected for analysis from each child. This segment length has been appropriate in our previous studies of EEG biomarkers for autism (36, 40) and epilepsy (43–45). Another study has demonstrated that continuous 20 s segments of EEG recording are sufficient for analysis of cognitive function with nonlinear measures (51), and other studies have found that EEG segments of 60 s or less are stable for nonlinear analysis (58). The steps used to process EEG recordings, extract latent features, and predict or detect outcomes are shown in Figure 1. Each step shown (1, 2, 3a, and 3b) is described in detail below.

**Figure 2:**
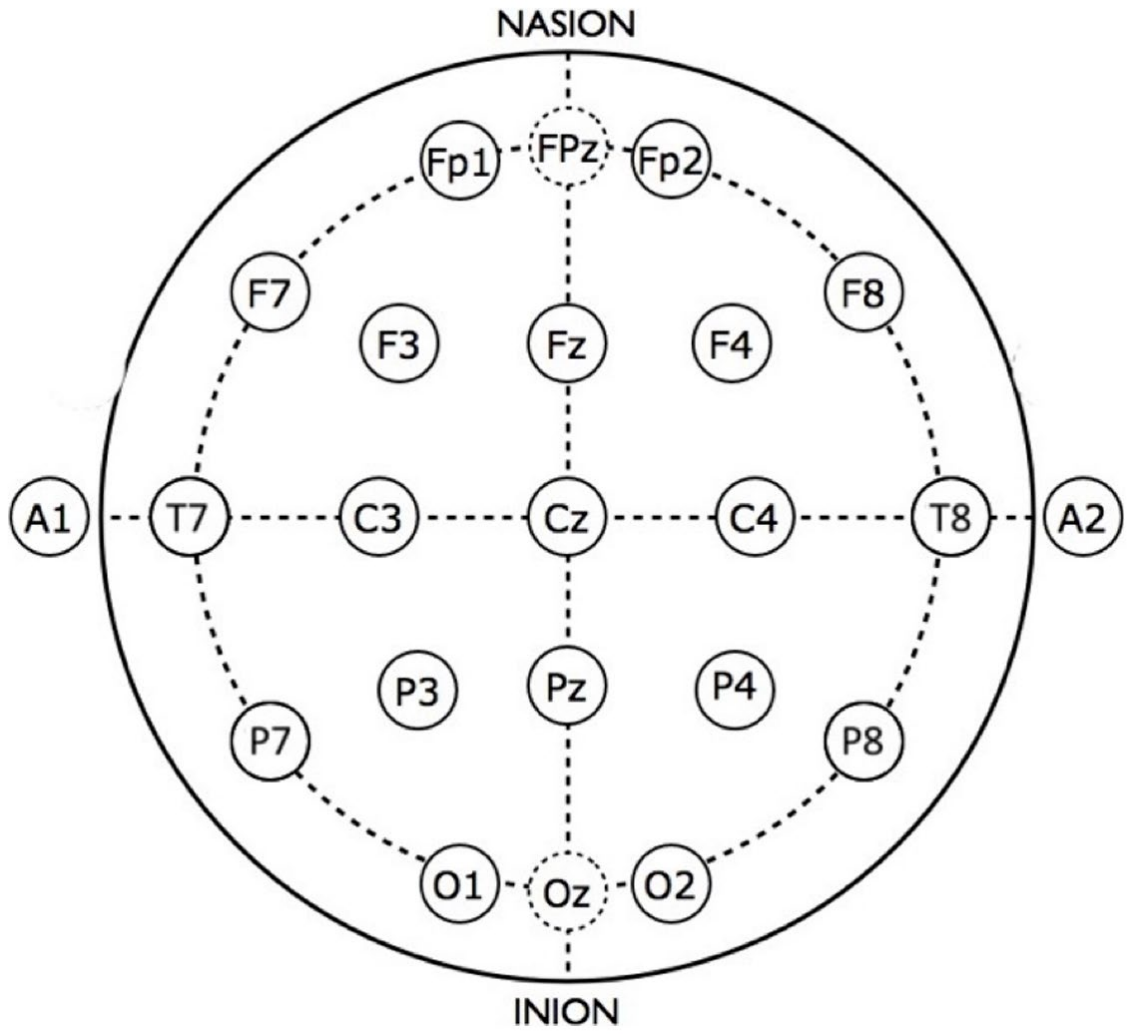
Scalp view of sensor locations for the standard 10–20 EEG montage.

The EEG signal from each of 19 electrodes corresponding to the traditional 10–20 arrangement were selected from the 128-channel Sensor Net dataset as shown in Figure 2. Each signal was decomposed into power-of-two frequency bands using the Daubechies (DB4) wavelet transform, which has been found to be well-suited to EEG analysis (59). Wavelet details containing discrete frequency bands were reconstructed to yield discrete frequency sub-signals that correspond approximately to commonly used frequency bands: delta, theta, alpha, beta, gamma, and gamma+. Approximations from a wavelet transform have been shown (48) to be equivalent to “scales” used in multiscale entropy analysis (60); we choose to use wavelet details in order to adhere to traditional frequency bands.

A number of nonlinear values were computed from the EEG sub-bands with the goal of characterizing dynamical aspects of neural function as fully as possible. These include sample entropy (SampE), correlation dimension (CD), and detrended fluctuation analysis (DFA), all computed using the publicly available nolds package^1^. Additional nonlinear values were computed using Recurrence Quantitative Analysis (RQA). RQA, in principle, extracts all dynamical information about a dynamical system and works well even for short, noisy, non-stationary time series (61, 62). RQA measures were computed using the Python pyRQA package^2^ using the fixed RR option (RR = 0.05) and embedding dimension of 10. Values computed include determinism (DET), trapping time (TT), diagonal line length entropy (Lentr), maximum (Lmax), and mean (Lmean) (61–63).

A variation of recurrence plot analysis interprets the recurrence plot as a network. Recurrence network (RN) computation exploits an analogy between complex network theory and nonlinear time series analysis (64). This approach is complementary to RQA, resulting in additional nonlinear information that may not be extracted by previously discussed methods (32). In particular, the RN approach extracts information regarding the structure of the underlying chaotic attractors, which are unavailable using the conventional algorithmic methods of nonlinear time-series analysis (64). The public PyUnicorn package^3^ was used to compute two additional RN measures from each EEG time series that measures information loss on chaotic attractors, which may be directly relevant to pathological neurodevelopment (65). These include vertical entropy (VertEnt) and average white noise vertical length (AvgVertWhiteLen).

Recent research suggests that the brain is a complex dynamical system whose optimal computational performance occurs near the edge of chaos, a region midway between purely ordered behavior and completely random behavior (66). The relationship between age and EEG complexity has begun to be explored, but a complete investigation of EEG complexity and childhood neurodevelopment is needed. In very young infants, before and soon after birth, Sample entropy (SampEnt) has been found to increase prenatally, but near and soon after birth SampEnt vacillates (67). Approximate entropy has been found to be an accurate biomarker for age-related decline in older adults (68). The meaning of the physics context and mathematical descriptions of various complexity measures have made it difficult to understand how the complexity of EEG signals relates to behavioral constructs. A recent review attempts to provide an overview of this topic for neuroscientists and psychologists. In general, complexity measures “can be broadly categorized as measures of predictability and regularity” (69). It has been noted that “the present concept of entropy [in development] resembles the ‘entropy’ of physics and mathematical information theory only at the intuitive level” (70). Recently, the development of an approach to analyzing time series called reservoir computing exploits the information processing capability of complex dynamical systems (71–74). The implication is that physical and mathematical concepts from nonlinear or chaotic dynamics may be appropriate for describing the dynamics of brain function as computed from time series analysis. Although promising, a better understanding of the relationship among the brain, cognitive function, and dynamical systems concepts awaits explication. The behavioral symptoms that define psychiatric disorders may be characterized as higher levels of neural organization (70). In terms of dynamical systems, these higher levels of organization represent phase transitions in the system (75). In principle, the phase transitions that represent neural correlates of disordered behaviors, such as anxiety, should be reflected in measurable differences in nonlinear measures. We hypothesized that empirically comparing nonlinear measures in different clinical diagnostic groups (e.g., anxiety disorders, externalizing disorders, and healthy controls) would reveal these differences.

In summary, step 1 in Figure 1 represents decomposition of the time series from each EEG sensor into standard frequency bands. From each band, 12 nonlinear values were computed: SampE, CD, DFA, seven RQA values, and two recurrence network values. In principle, these nonlinear values give a relatively complete description of the dynamical function of the brain, as much as the imperfect scalp recordings will allow. Supplementary Table S1 lists the 12 nonlinear measures that were computed and gives a brief description of the formal meaning of each used in the time series analysis literature. We have also included a brief description about the possible neurophysiological meaning of each measure, although one of the goals for the latent feature extraction methods introduced in this paper was to begin to discover, empirically, the neuropsychiatric correlates of these measures. We speculate that these nonlinear measures may find a place in a matrix as fundamental neural measures that cut across multiple different diagnostic categories, much like in the Research Domain Criteria (RDoC) conceptualization of neuropathology (76, 77).

Nine of the measures used for our analysis were derived from recurrence plot analysis. Recurrence plots are two-dimensional projections of multidimensional phase portraits that result from an analysis of time series called time delay embedding. Recurrence plots result in intricate patterns that can be quantitatively analyzed to extract information about system dynamics (78). The idea for quantifying recurrence plots (RP) was first developed by Zbilut and Webber (79, 80) and extended with new measures of complexity (81). Measures based on diagonal structures in the RP are related to chaos-order transitions in systems dynamics (82), whereas measures based on vertical structures are related to chaos-chaos transitions or laminar phases (81). Although these concepts are well-defined mathematically for formal dynamical systems, their meaning in the context of cognitive neuroscience remains to be explicated. We posit that empirical studies, such as the current study, are one way forward to discovering the relations between dynamical measurements of the brain and observed behavior or clinical symptoms.

The scientific goal of our analysis was to find which of these sensors, frequency bands, and nonlinear measures were most strongly associated with anxiety diagnosis. To accomplish this, we implemented and tested a novel approach to analyzing these complex measures based on supervised tensor factorization, which also enables a probabilistic output calculation that can be interpreted as a risk factor. To test our algorithm, we first applied supervised tensor factorization to find latent EEG features associated with calendar age. Given that development may be considered a process of brain complexification, we hypothesized that latent complexity features would be strongly correlated with age and thus provide a readily understood application of this approach to EEG analysis.

#### 2.2.2. Supervised tensor analysis for latent feature discovery

A significant challenge with multiscale nonlinear signal analysis is finding latent features among the large number of computed values across all scalp sensors, frequencies, and nonlinear measures. We used a novel approach to organizing EEG measures based on a multiarray or tensor (83). Tensor factorization extends to higher dimensions common matrix factorization methods, such as principal components analysis (PCA) and the singular value decomposition (83). Recently, supervised tensor decomposition methods have been developed that allow labeled data plus covariate data to be used to condition the selection of underlying latent patterns. We adopted one such approach, based on a supervised version of the Canonical Polyadic (CP) approach to tensor decomposition (54).

**Figure 3:**
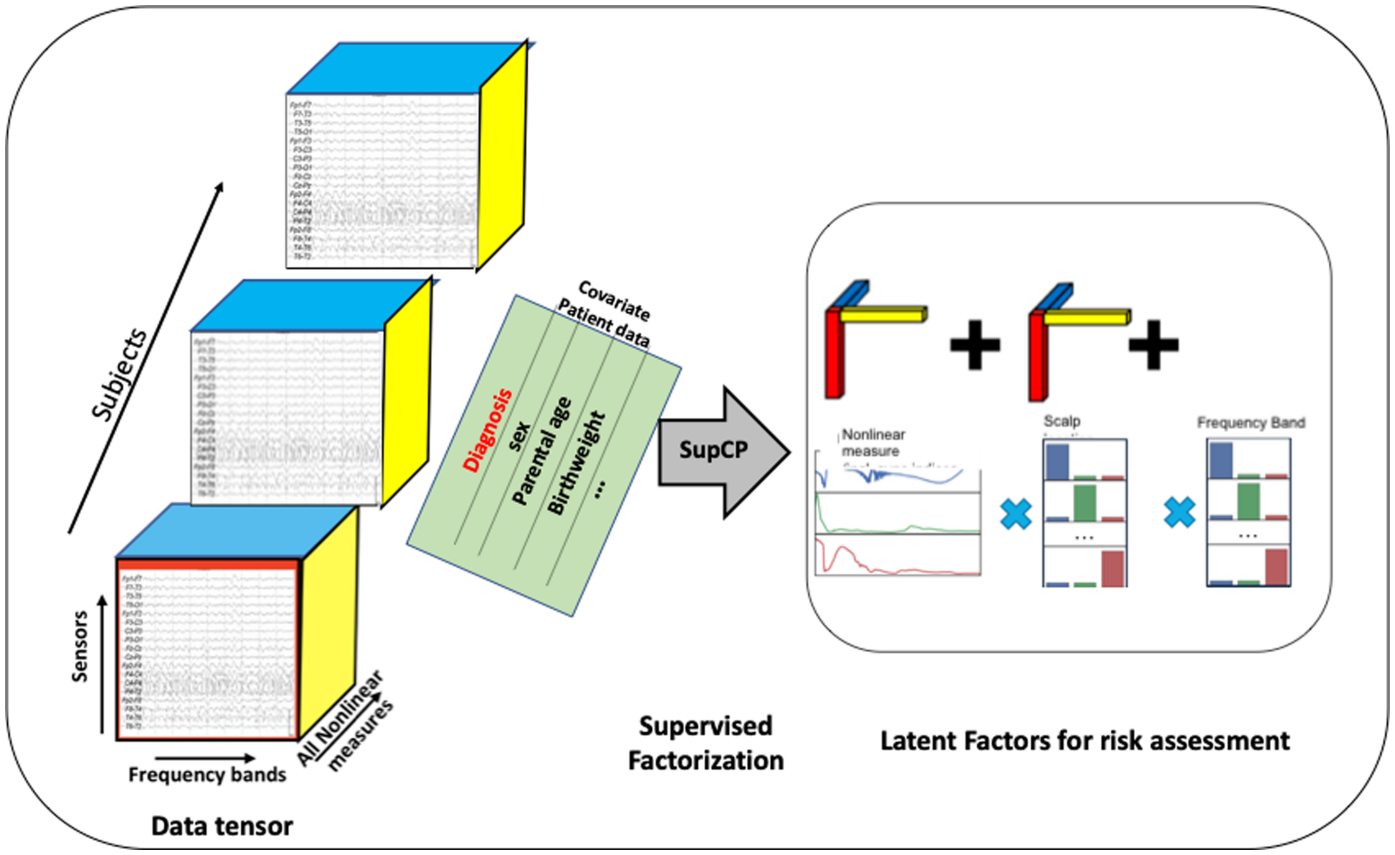
Tensor organization of multiscale EEG data with additional covariate data shown as a matrix sharing a single axis, participant ID, with the EEG tensor. The developmental time axis is not shown. Latent factors can be used as input to traditional machine learning algorithms. Alternatively, a probabilistic outcome can be computed using the tensor model.

As described previously, each EEG recording consisted of time series derived from each of the 19 scalp sensors. Each of these time series was decomposed into several sub-bands corresponding to the commonly used bands of delta, theta, alpha, beta, and gamma, and on each sub-band the nonlinear measures were computed. These computed values were arranged into a 3-dimensional tensor as shown in Figure 3. An additional axis for time, which is not shown in the figure, was included for recordings taken at successive ages within child. A data tensor was derived for each child, comprising the first dimension, shown as the succession of boxes in Figure 3.

When using this approach, the number of desired latent factors must be specified as the tensor rank *a priori*. There is no precise formula for tensor rank determination (54); thus, it is often determined experimentally (83). We found that choosing a rank greater than 30 did not improve results. Hence, *R* = 30 is used throughout the remainder of our analysis.

Similar to the Singular Value Decomposition (SVD) or principal components analysis (PCA) for matrices, the tensor decomposition will find a reduced dimensionality, latent feature set (84–86). The canonical polyadic decomposition (CPD) of a rank R tensor factorizes the tensor as a sum of R rank-1 tensors (83), as illustrated in Figure 3. Our approach is based on a *supervised* CP method, SupCP, that uses the specific class labels and covariate data to extract features that are specifically suited to the particular classification task at hand (54). The result is a set of latent factors that are a linear combination of the most significant contributions in each tensor dimension. We emphasize here that in the SupCP factorization algorithm, the latent variables are informed by both group labels and additional covariates. The labels and optional additional covariate data enable computation of latent structures that are more relevant to the groups of interest and interpretable due to covariate supervision (54). The side matrix in Figure 3 that is aligned with the primary data tensor represents the group labels and covariate data.

The extracted latent factors were used in one of two ways for classification and regression: (1) the extracted features were used in traditional machine learning methods, which we applied to test the method for predicting calendar age from nonlinear values. Latent features were input to several different regressors available in the Python scikit-learn package.^4^ Classification can also be achieved using similar classifiers when the outcome labels are discrete variables, such as having an anxiety disorder or not. This approach to detecting anxiety disorder diagnosis was used. (2) A probability of class membership can also be computed directly using the supervised factorization. Given an EEG tensor for participant *X*_i_, the likelihood of *X*_i_ belonging to each of the classes is denoted *P*(*X*_i_,|Y*), where Y* could give the specified class and can also include the covariate data for the participant of interest. The likelihood was computed by taking the exponent of Eq. (4) in (54), which gave the log-likelihood, marginalized over U. Given a prior probability for each class [e.g., *P*(*Y* = *k*)], Bayes rule was applied to compute the posterior probabilities for *X*_i_ belonging to each class, for example, *P*(*Y*_i_ = *k*|*X*_i_). The class with the highest membership probability was then chosen as the predicted label. The probability may also be interpreted as a risk probability, although we have not explored such an approach in the current analysis. The result of this computation was to assign a most likely class membership (healthy control or disorder) for each member of the dataset. Group statistics were then computed for the model as an initial assessment of the latent feature model. Future studies with independent test data will be required to test model generalizability.

The raw data included EEG recordings in infancy, and at 3, 5, and 7 years of age. Sociodemographic data included the exact age when the first EEG recording was made in the first year, the child’s sex assigned at birth, birthweight, race, ethnicity, and parental education. The current implementation of SupCP used in this paper (54) assumes that the axis scales have discrete values, such as frequency band and sensor location. In our use of the algorithm for anxiety and externalizing disorder biomarker discovery, we used integer values for the EEG recording age: 1 (infancy), 3 years, 5 years, and 7 years, when the actual recording age in months was available and varied slightly around the target age.

#### 2.2.3. Child psychiatric diagnoses

Child lifetime psychiatric history was assessed using the Diagnostic Interview for the Preschool Age (DIPA). The DIPA is a validated semi-structured interview designed specifically for assessing DSM diagnoses in young children (87). Version 7/12/14 of the DIPA was administered, which reflects all relevant DSM-5 criteria. Trained, clinically supervised research staff administered the DIPA to the child’s mother in person or over a HIPAA-compliant video conferencing platform when the child was approximately 5 years of age. The current analyses considered psychiatric disorders assessed via the following modules: generalized Anxiety Disorder (GAD), Separation Anxiety Disorder (SAD), Social Phobia/Social Anxiety Disorder (SP), Attention-Deficit/Hyperactivity Disorder (ADHD), Oppositional Defiant Disorder (ODD), Conduct Disorder (CD), Major Depressive Disorder (MDD), Disruptive Mood Dysregulation Disorder (DMDD), Obsessive–Compulsive Disorder (OCD), and Posttraumatic Stress Disorder (PTSD). Symptom frequency and severity and functional impairment were assessed for each module to determine if diagnostic criteria were met during the child’s lifetime. For the purposes of the current analyses, children were categorized by their lifetime diagnostic history: (1) anxiety group = presence of at least one anxiety disorder, including GAD, SAD, and/or SP, with no history of an externalizing disorder, (2) externalizing group = presence of ADHD, ODD, and/or CD, with no history of an anxiety disorder, and (3) healthy control group = no history of any psychiatric disorder, i.e., none of the diagnoses listed above. Children who were comorbid for both an anxiety disorder and an externalizing disorder were not included in analyses to allow for comparison of distinct anxiety versus externalizing groups.

#### 2.2.4. Covariates

Covariates considered included child’s age, sex assigned at birth, race, ethnicity, and maternal and paternal educational attainment. Child age was calculated as a continuous variable. The child age at initial EEG recording was included as covariate information when analyzing the latent EEG features for anxiety or externalizing disorders. For age regression analyses, the exact age at each recording was used as the predicted outcome value. Child race was categorized as American Indian/Alaska Native, Asian, Native Hawaiian/Other Pacific Islander, Black/African American, White, or more than one race. Child ethnicity was categorized as Hispanic/Latino or not Hispanic/Latino. Parental educational attainment was categorized as less than high school degree, high school degree/GED, Associate’s degree, Bachelor’s degree, Master’s degree, or graduate degree (M.D., Ph.D., J.D., or equivalent). Race, ethnicity, and parental education were initially evaluated as covariates, but found to not improve accuracy of any results. Hence, these variables are not considered further in this study.

## 3. Results

We first review sociodemographic characteristics of our sample, then present the results from our test case of predicting calendar age from latent EEG features derived using supervised tensor factorization. Finally, we present the main results from our analysis of clinical diagnosis detection or prediction.

### 3.1. Sociodemographic analysis

Table 1 provides the sociodemographic characteristics of the study sample. Due to exclusionary criteria, all children were born full term (37 weeks to 43 weeks). Children were also primarily of birthweight appropriate for gestational age (M = 3,541 g, SD = 701 g, 96% > 2,500 g). As noted above, children were excluded from the study if there were any known developmental delays, neurological or neurodevelopmental disorder or trauma, or maternal use of certain medications during pregnancy. At the time of these analyses, EEG data were available for 150 children in infancy, 109 at age 3 years, 114 at age 5 years, and 34 at age 7 years.

**Table 1:** Sample characteristics (*N* = 150).

	*n*	%	M	SD
Child age, infant assessment (months)			7.92	2.75
Child age, 3 years assessment (months)			37.52	1.46
Child age, 5 years assessment (months)			62.43	1.80
Child age, 7 years assessment (months)			88.84	3.16
Child sex assigned at birth (male)	76	50.7		
Child race				
White	122	81.3		
Asian	4	2.7		
Black/African American	3	2.0		
More than one race	20	13.3		
Child ethnicity (Hispanic)	20	13.3		
Maternal age at enrollment (years)			33.85	3.67
Paternal age at enrollment (years)			35.82	4.58
Maternal education				
Associate’s degree	8	5.3		
College degree	40	26.7		
Master’s degree	68	45.3		
Graduate degree	33	22.0		
Paternal education				
Associate’s degree or less	23	15.3		
College degree	45	30.0		
Master’s degree	50	33.3		
Graduate degree	30	20.0		
Annual household income				
<$35,000	3	2.4		
$35,000–$49,999	5	3.9		
$50,000–$74,999	14	10.9		
$75,000–$99,999	23	17.8		
$100,000+	84	65.1		

EEG data were available for 150 participants in infancy, 109 at age 3 years, 114 at age 5 years, and 34 at age 7 years. Missing data for remaining sociodemographic variables as follows: child sex assigned at birth = 0, child race = 1, child ethnicity = 2, maternal age at enrollment = 3, paternal age at enrollment = 7, maternal education = 1, paternal education = 2, annual household income = 21.

As displayed in Table 2, 74% (*n* = 111) of the sample did not meet criteria for any of the assessed clinical disorders [healthy control (HC) group], 19% (*n* = 29) met criteria for one or more anxiety disorders and no externalizing disorder [anxiety disorder (AD) group], and 7% (*n* = 10) met criteria for one or more externalizing disorders and no anxiety disorder [externalizing disorder (ED) group]. One child in the externalizing group had a comorbid MDD diagnosis. No child met criteria for OCD. Two children had a history of both anxiety disorder(s) and externalizing disorder(s), and one child met criteria for PTSD and no other diagnoses and thus did not fit into any of the *a priori* diagnostic groups. These three children were not included in the final sample size of 150. Among the 29 children in the anxiety disorder group, 25 (86%) had met criteria for one anxiety disorder and 4 (14%) for two anxiety disorders. As shown in Table 2, the most frequently represented diagnosis was SP, followed by SAD, and then GAD. Among the 10 children in the externalizing disorder group, 7 (70%) met criteria for one externalizing disorder and 3 (30%) for two externalizing disorders. ADHD was the most commonly represented diagnosis, followed by ODD, and one instance of CD. No differences were found among the three diagnostic groups on any of the covariates, including sex assigned at birth, birthweight, or maternal or paternal education level. Multiple comparison among the diagnostic groups for each covariate was computed using the anova function and TukeyHSD function in R to show *p*-values for each pairwise comparison: *p* > 0.2 in all cases.

**Table 2:** Child lifetime psychiatric diagnostic group by age 5 years.

Diagnostic group	*n*	% within sample with diagnosis	% within group with diagnosis
Healthy control (no disorder)	111	74%	–
Anxiety disorder(s) only	29	19%	
Social Phobia/Social Anxiety Disorder	19	13%	66%
Separation Anxiety Disorder	10	7%	34%
Generalized Anxiety Disorder	4	3%	14%
Externalizing disorder(s) only	10	7%	
Attention-Deficit/Hyperactivity Disorder	8	5%	80%
Oppositional Defiant Disorder	4	3%	40%
Conduct Disorder	1	1%	10%

Diagnoses within diagnostic group (anxiety; externalizing) are not mutually exclusive. Children comorbid for one or more anxiety disorders and one or more externalizing disorders (*n* = 2) were not included in analyses.

### 3.2. Developmental age biomarker from nonlinear EEG analysis

**Figure 4:**
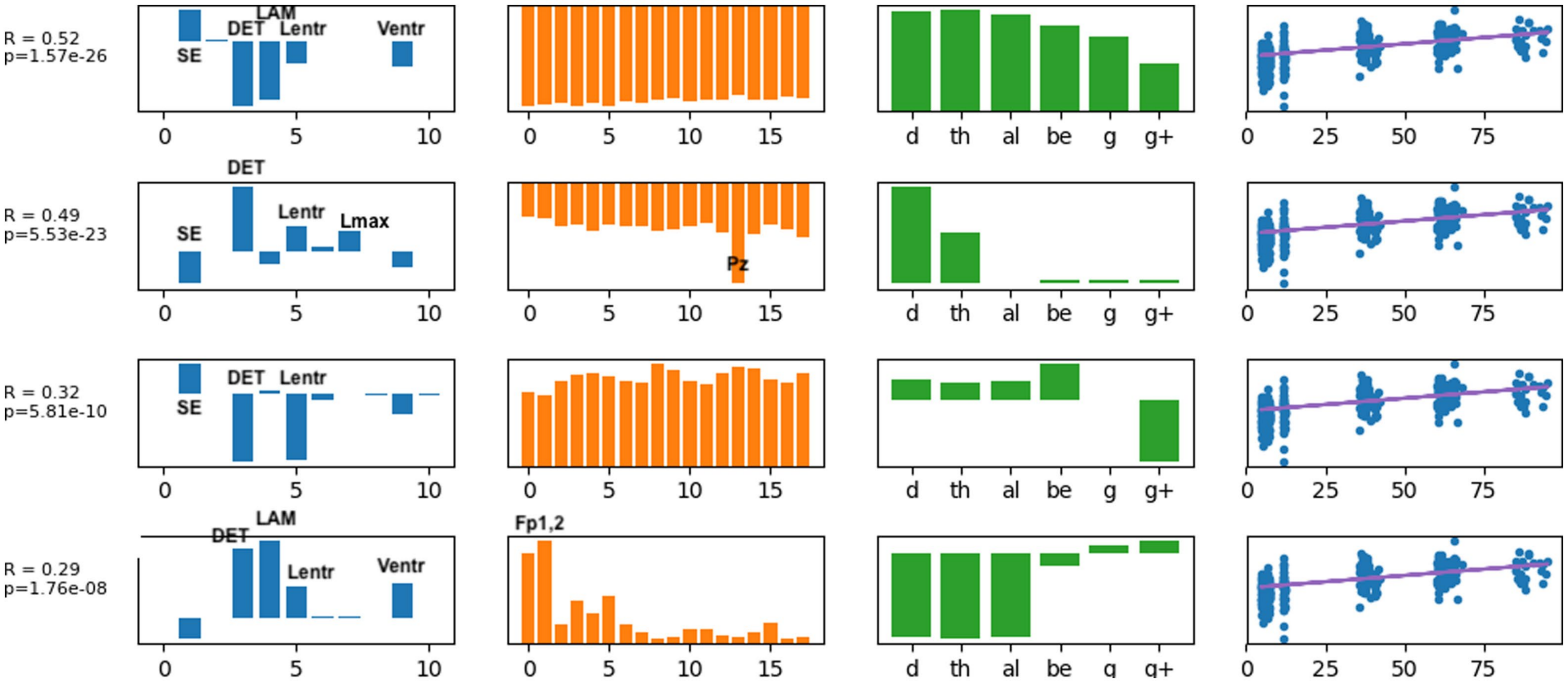
The latent factors that had an individual correlation with age of |*r*| > 0.2 are displayed here as the most informative factors for age. The right-most column illustrates age correlation with the *single latent feature* for that row. Age correlation results in Table 3 were computed using all latent features shown here.

Typical brain development may be considered a process of complexification and refinement (88). If so, measures of electrodynamical complexity would be expected to change with age. A search of hundreds of computed values is not an efficient way to identify biomarkers, nor is it correct to assume that these values are independent. Some nonlinear values may measure similar properties, such as sample entropy and the entropy derived from recurrence plots. Treating all measures as independent variables may miss interactions between variables when correlating with age and is a time-consuming process. As described in the Methods section, EEG features were organized into a tensor structure for analysis. We sought to discover latent variables, which combine nonlinear measures, scalp locations, and frequency ranges, that were most robustly associated with age using supervised tensor factorization to extract latent factors. Age was the only covariate used for the supervised factorization calculation. The latent factors were then used to compute correlations with age. Thirty latent features were computed. Each latent variable can be written as a linear set of weights for the features in each axis, represented by each column in Figure 4. Thus, each factor is represented by a vector of weights. A correlation coefficient and value of *p* were computed for each latent factor, and the individual factors were ranked from largest correlation coefficient to smallest. All latent factors with a correlation coefficient |*r*| > 0.2, selected from the 30 latent features that were computed, are plotted in Figure 4. A complete table of all nonlinear values computed is presented in Supplementary Table S1.

Several results are found from examining the latent features in Figure 4. Determinism (DET) was consistently the most highly weighted measure in all latent factors. Laminarity (LAM) and the three entropy measures Lentr, SampE, and VertEnt were also represented in the latent factors. We have not done further research to determine if these entropy measures are independent or themselves highly correlated. They may be different algorithms for a similar measure of signal complexity. Lmean, TT, and AvgWhiteVertLen had very low weight across all frequency bands and all scalp locations, indicating that these did not contribute to age predictions. Frontal and central-parietal scalp electrodes and lower frequencies were found to be the most important factors associated with age.

We then used the three latent factors shown in Figure 4 as predictors for several different machine learning regression methods and evaluated the models using 5-fold cross-validation. Similar regression coefficients were found for all methods. Results are shown in Table 3. We chose to use latent factors that individually had at least weak correlation (|*r*| > 0.2) with age. Using more latent factors did not greatly improve correlation, suggesting that the highest ranked latent features contain most of the predictive information. The relatively similar correlation coefficients for all methods suggest that the latent variables are highly correlated with age, independent of the regression method. The best results were found with the random forest regressor, *r* = 0.76, *p* = 7.6e-72, and the mean age error was 16.7 months.

**Table 3:** Coefficients of determination and correlation coefficients computed from latent factors extracted by SupCP using calendar age as the predicted outcome.

Regression model	Coefficient of determination	Correlation coefficient
KNN	0.58	0.76
Linear regression	0.52	0.72
Random Forest	0.58	0.76
Gradient Boost	0.54	0.74
SVM rbf	0.59	0.77
SVM linear	0.46	0.67

The three highest ranked latent features shown in Figure 4 were used as input to machine learning regression algorithms in a 5-fold cross validation scheme. Coefficients of determination and correlation coefficients were computed for each participant, with results as shown. Increasing the number of latent features did not improve the correlation coefficient.

### 3.3. Biomarkers for anxiety and externalizing disorders

Potential biomarker profiles for anxiety disorders and for externalizing disorders were found by extracting latent features from nonlinear EEG measures, using the probabilistic classification denoted in step 3b in Figure 1. A 5-fold cross-validation scheme was used to test the generalizability of the latent features (or, *n*-fold cross-validation when the number of participants in the least populated group, *n*, is less than 5). The following comparisons were conducted independently: anxiety disorders versus healthy controls; externalizing disorders versus healthy controls; and anxiety disorders versus externalizing disorders. For each comparison, a stratified training/test set split was computed using the StratifiedKFold function in the Python scikit-learn package. The training set for each fold was used to compute latent features via the supervised tensor factorization algorithm SupCP (54). Using the latent features derived from the training set, the probability of class membership was computed for the test set. In this way, a predicted class membership was computed for every child using latent features that were derived from independent training data. The outcome probabilities were used to compute a Brier score and, by varying the threshold for class membership, the area under the receiver operator characteristic (AU-ROC) curve. The resulting scores are presented in Table 4.

**Table 4:** Five-fold cross-validation was used to compute 30 latent features using the SupCP tensor factorization algorithm.

Age (*N* con, *N* disorder)	AU ROC, Brier score		
	EEG only	EEG + sex	EEG + bw + sex
Anxiety disorders versus healthy controls			
1 (103, 27)	0.30, 0.73	0.42, 0.51	0.47, 0.30
3 (63, 17)	0.44, 0.21	0.44, 0.21	0.53, 0.21
5 (72, 22)	0.48, 0.60	0.45, 0.47	0.54, 0.28
7 (20, 7)	0.63, 0.25	0.76, 0.26	0.72, 0.29
1,3 (63, 16)	0.37, 0.72	0.41, 0.45	0.51, 0.29
3, 5 (49, 14)	0.58, 0.31	0.55, 0.23	0.55, 0.26
5, 7 (17, 6)	0.60, 0.34	0.70, 0.28	0.68, 0.24
1, 3, 5 (49, 14)	0.44, 0.69	0.38, 0.51	0.53, 0.26
3, 5, 7 (14, 4)	0.86, 0.21	0.82, 0.08	0.57, 0.22
1, 3, 5, 7 (14, 4)	0.64, 0.19	0.46, 0.23	0.50, 0.28
Externalizing disorders versus healthy controls			
1 (103, 9)	0.33, 0.67	0.52, 0.43	0.24, 0.22
3 (68, 6)	0.68, 0.36	0.69, 0.24	0.58, 0.13
5 (72, 8)	0.61, 0.33	0.60, 0.25	0.59, 0.11
1,3 (63, 5)	0.78, 0.60	0.65, 0.14	0.71, 0.36
3, 5 (51,5)	0.57, 0.26	0.82, 0.16	0.84, 0.09
1, 3, and 5 (51, 5)	0.63, 0.44	0.79, 0.24	0.70, 0.07
Anxiety disorders versus externalizing disorders			
1 (103, 36)	0.29, 0.71	0.41, 0.52	0.51, 0.30
3 (63,21)	0.44, 0.31	0.64, 0.26	0.71, 0.19
5 (22,8)	0.51, 0.29	0.63, 0.25	0.50, 0.31
1, 3 (63, 21)	0.48, 0.69	0.48, 0.51	0.59, 0.27
3, 5 (14,4)	0.45, 0.27	0.75, 0.30	0.86, 0.19
1, 3, and 5 (14, 4)	0.68, 0.29	0.79, 0.23	0.68, 0.22

Using latent features from each training set, the probability of belonging to either of two comparison groups (anxiety disorder, externalizing disorder, or healthy controls) was computed. The Brier scores were then computed from these probabilities. The area under the receiver operator characteristic (AU-ROC) curve was computed by varying the probability threshold for group membership. The yellow shaded boxes are the best scores for each classification. Gray shaded boxes are also considered to demonstrate some predictive value, based on the guide that AU-ROC >0.70 is acceptable and >0.80 is considered good (89). We also note that the Brier score generally follows the AU-ROC curve in this table. Brier score <0.20 indicates modest predictive ability and <0.10 is considered good (90).

The supCP algorithm allows for covariates to be included as training data along with the class labels. We evaluated available covariate data to determine if any improved predictive results, including child sex assigned at birth, birthweight, race, ethnicity, and maternal and paternal education. We found that sex assigned at birth had the largest influence on results. Both anxiety and externalizing disorder prediction accuracy were better when sex assigned at birth was included a covariate. Including birthweight as a covariate degraded accuracy for anxiety but resulted in modest improvement to prediction of externalizing disorders. As noted above, none of the other covariates appeared to improve results and were thus not examined further. The following results are inferred from Table 4.

1. None of the three comparison groups (anxiety, externalizing, and healthy controls) could be distinguished using EEG data alone from a single recording; multiple years or sex as a covariate were required. Infancy data were not useful for improving any results.
2. Anxiety disorders were detected using 3-, 5-, and 7 years data with only EEG features or, with better results, when sex assigned at birth was included as a covariate. Although 7 year data alone produced better results than any combination of earlier (1-, 3-, and 5 years) data, it appears that a trajectory (3-, 5-, 7 years) gave better results than a single snapshot.
3. Externalizing disorders were detected with 3- and 5 years EEG data if sex assigned at birth was included as a covariate. When birthweight was included as an additional covariate, results improved slightly. As noted above, there were no group differences among diagnostic groups in birthweight; thus, birthweight alone could not differentiate among the groups. Inclusion of infancy EEG data with 3- and 5 years EEG data appeared to degrade results slightly, suggesting that most of the information came from 3- and 5 years data and that including infancy data changed the (linear) trajectory.
4. Anxiety and externalizing disorders were distinguished from each other by 3- and 5 years data if sex assigned at birth and birthweight were also considered. No 7 years EEG recordings were available at the time of this study for children with externalizing disorders to be able to consider the contribution of 7 years EEG data.
5. If anxiety and externalizing disorders were combined together into a single “atypical” group, classification was poor (AU ROC = 0.53, Brier = 0.42 when 3- and 5 years EEG data and sex at birth were included). This finding suggests that different latent factors are needed to distinguish anxiety vs. externalizing disorders from healthy controls. These results are not shown in Table 4.

For both anxiety and externalizing disorders groups, changes over two or more measurements appear to be more informative than any single snapshot of brain activity. This suggests that the trajectory of nonlinear values is essential to detecting both anxiety and externalizing disorders. Compared to anxiety disorders, which required 7 years data to distinguish from healthy controls, externalizing disorders were detected earlier, at 3 to 5 years. Overall, inclusion of infancy EEG data does not appear to contribute to detecting externalizing disorders when comparing to either controls or children with anxiety disorders.

**Figure 5:**
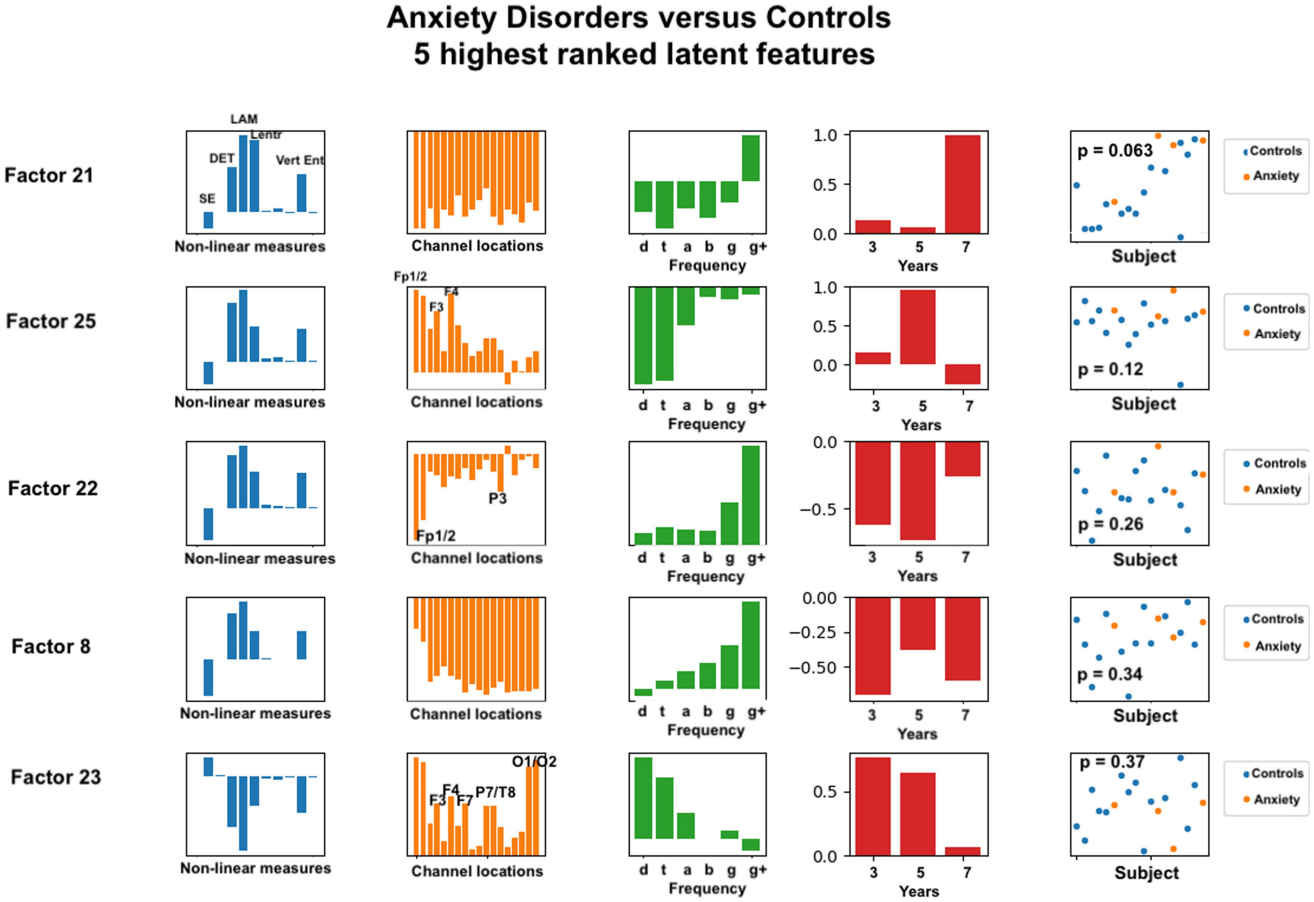
Latent factors extracted for distinguishing anxiety disorder group from healthy control group. Covariates were age of initial EEG measurement and sex assigned at birth. The right-most column shows the distribution of weights for the single latent factor in that row. That is, it is a visual illustration of the contribution of that factor. *p*-values for the factor are also given for each factor alone. The contribution of each nonlinear measure, sensor location, and age to the latent factors can be seen in the bar charts in each column. The dots in the right-most column show separation of diagnostic groups using the single latent variable represented by that row.

**Figure 6:**
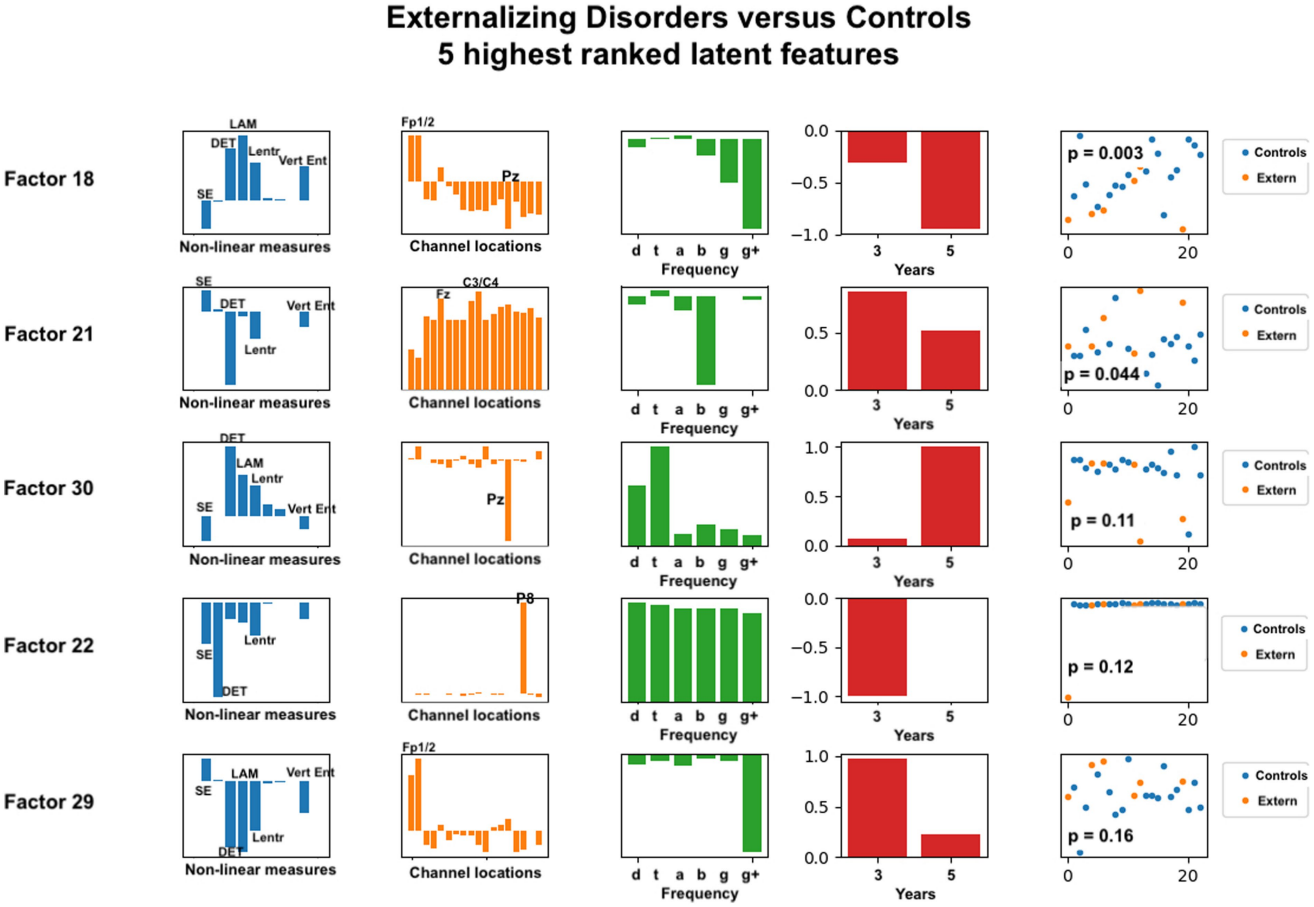
Latent factors extracted for distinguishing externalizing disorder group from healthy control group. Covariates were age of initial EEG measurement and sex assigned at birth. The right-most column shows the distribution of weights for the single latent factor in that row. That is, it is a visual illustration of the contribution of that factor. *p*-values for the factor are also given for each factor alone. The contribution of each nonlinear measure, sensor location, and age to the latent factors can be seen in the bar charts in each column. The dots in the right-most column show separation of diagnostic groups using the single latent variable represented by that row.

The latent features that were extracted from the EEG tensor for each of the three group comparisons were also computed from the full dataset (not using cross validation) in order to examine which nonlinear measures, sensors, and frequencies were contributing the most to the latent features. This information is shown in Figures 5, 6 for anxiety disorders and externalizing disorders, respectively. For Figure 5 (anxiety), we used the data that gave the best classification results: 3-, 5-, and 7 years EEG data plus sex at birth as a covariate. For Figure 6 (externalizing disorders), we used 3- and 5 years EEG data plus sex at birth as a covariate because these data also gave good classification results and used the same variables as for anxiety disorders (except for 7 years EEG data, which were not available for children with externalizing disorders). This approach allows the respective latent factors to be compared most easily. Inclusion of birthweight had minor effect on the results and, upon visual inspection, did not change the latent factor results for externalizing disorders; thus, showing additional figures would not convey more information about the latent factors.

Each row in the figures represents a single latent factor. The latent factors were ranked by extracting the weight of that latent factor for each participant [weights are computed in the factorization algorithm and reported in the U matrix, as described in (54)]. Although 30 latent features were computed, we reasoned that features that individually showed the greatest differences between healthy controls and either of the diagnostic groups would contribute most toward differentiating the groups. This reasoning is consistent with our findings for age correlation that used only the highest ranked features for correlation analysis. Our primary goal was to identify the set of latent variables that contribute the most to classification accuracy. Each row of each figure shows the relative weight or contribution from elements of each axis of the data tensor: nonlinear measure, sensor location, frequency band, and developmental age at which the EEG recording was done.

For anxiety disorders, the nonlinear features most highly weighted were laminarity (LAM), determinism (DET), and the entropy measures (SampE, Lentr, and VertEnt). These measures were consistently the dominant contributors, in this order. A brief discussion of these variables was given in the Methods section. Laminarity is related to the amount of laminar or smooth phases in the system and intermittency or alternation between periodic and chaotic regimes, whereas determinism is related to dynamical system predictability. The entropy values are all measures of signal complexity, reflecting complexity of the neural circuits that created the signal. A lower value of sample entropy indicates more self-similarity in a signal and lower complexity. We did not test how highly correlated these different entropy measures are.

Figure 5 also reveals that, for anxiety disorders, frontal sensors, followed by parietal sensors, are more heavily weighted, although all sensors contributed to the latent factors. Latent factors appeared to be composed primarily of either lowest (delta, theta) or highest frequencies (gamma, gamma+), but not both. Middle frequencies are weighted less. Data from 3, 5, and 7 years appear to contribute to anxiety latent factors, with the two most significant latent factors composed of only 7 years and only 5 years data, respectively.

The composition of latent factors for externalizing disorders reveals some differences from those for anxiety disorders. Although laminarity, determinism, and the three entropy measures are again most prominent, their ratios appear to vary considerable in each of the latent factors. The most prominent sensor locations are three frontal locations, Fp1, Fp2, and Fz, and two parietal locations, Pz and P8. All other sensors are less prominent than they were for anxiety disorders. As for anxiety disorders, latent factors for externalizing disorders tended to be composed of either low or high frequency bands, not both, although one latent factor was composed of all frequencies equally. Data from 3 years and from 5 years appeared equally represented.

## 4. Discussion

The overall objective of the current study was to compute a set of nonlinear measures across traditional frequency bands from EEG data assessed repeatedly from infancy to middle childhood and then use a supervised tensor factorization approach to extract latent features from the nonlinear measures that were associated with childhood anxiety diagnosis, the most common psychiatric diagnosis in children as well as adults. To test for specificity of any findings in relation to anxiety disorder diagnosis (versus being an indicator of general psychopathology risk), we extracted latent features that were associated with externalizing diagnosis and compared them to those associated with anxiety diagnosis. We also combined the two diagnostic classes into a single ‘atypical’ class and compared to a healthy control group. To demonstrate the use of tensor factorization to extract latent features, we first applied this method to predict child calendar age.

### 4.1. Neurodevelopmental age

We found that the correlation between calendar age and EEG signal complexity is strong, supporting the validity of our approach. As described in our Introduction, the primary reason for correlating nonlinear measures with calendar age at this time was to demonstrate the validity of supervised tensor factorization, not primarily to predict age or cognitive development. Nevertheless, this methodology might be useful for more extensive analysis of EEG features for gauging development. For future analyses, there may be more appropriate developmental targets than calendar age to consider in relation to EEG signal complexity. Children’s brains develop at different rates and have different capacities at different times. Future studies may focus on finding relations between EEG features, ideally with clinical covariates such as sex assigned at birth, family history, and/or genetics, and specific developmental cognitive assessments. Digital biomarkers that provide insight into a child’s developmental trajectory based on nonlinear EEG features and relevant clinical data could be valuable both for monitoring typical development and for detecting the first signs of deviation, allowing a risk profile to be computed for each of several different disorders. Such approaches may provide critical information for the early identification of vulnerable children, allowing application of prevention efforts that optimize ultimate development and functioning.

### 4.2. Anxiety disorders and externalizing disorders

For anxiety disorders, five nonlinear measures appeared to be prominent in the same ratios for all of the highest ranked factors. Laminarity was consistently highest, followed by determinism, and then the three entropy measures. We found that frontal electrode locations (Fp1, Fp2, F3, F4, and Fz) were most prominent, while parietal and occipital (P3, P7, T8, O1, and O2) were slightly more prominent than the remaining electrode locations. Lower or higher, but not middle, frequency bands were associated with anxiety. This is consistent with previous findings that frontal and parietal EEG asymmetry are linked to anxiety disorders (24–26).

For externalizing disorders, the same nonlinear measures were most common in the latent factors, but their relative contributions were much more variable than was found for the anxiety group. Electrode locations revealed differences as well. Frontal electrodes (Fp1, Fp2) were clearly most prominent, with parietal electrodes Pz and P8 appearing dominant in two of the latent factors. The frequency contributions for externalizing disorders appeared more variable than for anxiety disorders, with all frequencies appearing across the five latent factors.

Also notable were the different patterns of age distributions for anxiety versus externalizing disorders. When compared to healthy controls, externalizing disorders could be detected using 3- and 5 years EEG data, whereas anxiety disorders required 7 years recordings, with findings most robust when 7 years data were considered along with 3- and 5 years data. Further, although birthweight alone was not significantly different among clinical groups, it may provide additional information to the algorithm that, combined with EEG measures, indicates a vulnerability specifically for externalizing disorders. In contrast, we found that detectable neural signals emerge later for anxiety disorders using our methods. The current study used EEG data collected during a neutral resting state. EEG data collected while the child is exposed to certain emotional stimuli particularly relevant to anxiety (e.g., threatening stimuli, such as angry or fearful faces) may prove more informative for earlier prediction of anxiety risk and should be tested in future studies.

For both anxiety and externalizing disorders, including infant EEG data did not improve group prediction accuracy. This may suggest for both disorders that EEG-derived biomarkers may be measuring actual brain changes associated with the emerging disorder rather than early prodromal changes that lead to later symptoms emerging. This finding contrasts with our previous research on EEG biomarkers for autism spectrum disorder, which found that very early (3 to 9 month) EEG data predicted a later outcome that was diagnosed at 3 years of age (36). Alternatively, as suggested above, infant EEG data may contribute to the prediction of psychopathology risk if collected under certain conditions (e.g., during exposure to emotional stimuli). Our results also may indicate that resting state EEG analysis, which is predicated on measuring stable states of the brain as a dynamical system, does not detect the earliest changes occurring in infancy that precede establishment of anxiety-exhibiting neural circuits. Finally, our EEG measures in infancy may prove to be useful in predicting later anxiety when combined with other variables not included in the current analysis (e.g., caregiving environment, temperament). We plan to explore this line of research in future work to elucidate the specific combination of data needed to optimize prediction of anxiety risk as early in development as possible.

Future studies that collect repeated measures of EEG might create developmental trajectory models, using diagnostic status to distinguish trajectories for different disorders from healthy controls. This could allow a more definitive examination of when EEG signal trajectories diverge in relation to clinical symptoms and the relative value of timing of EEG assessment and trajectory information to the prediction of particular diagnoses. Further, assessing relevant environmental factors, such as stress exposures and qualities of the caregiving environment, and including these as covariates in the model would help elucidate the role of the environment in shaping neural trajectories toward different clinical diagnoses.

Latent variables derived from nonlinear EEG measures alone (i.e., with no consideration of covariates) were able to differentiate children with one or more anxiety disorders from healthy controls. Inclusion of sex assigned at birth as a covariate improved accuracy as measured via AU ROC and the Brier score (0.21 to 0.08). Inclusion of birthweight as a covariate degraded detection of anxiety disorders markedly, suggesting it is irrelevant to anxiety development, at least when birthweight is within normal range as in this sample, and interferes with the risk probability calculation. Algorithm improvement should enable extraneous information like this to be given little weight and thus largely excluded in probability calculations. That is, although covariates were included in the feature selection algorithm, the covariates themselves were not included as weighted latent factors. We speculate that if fully coupled tensor + covariate factorization was used (planned future work), then birthweight would receive little weight and thus not affect prediction results. As used now, the covariates are included in the final probability predictions, demonstrating that birthweight negatively affected anxiety detection.

Our findings suggest that covariate participant data may be essential context for interpreting functional measurements using nonlinear EEG analysis. Although birthweight did not differ among our three clinical groups (anxiety disorder, externalizing disorder, healthy controls), this variable made a modest difference in the ability of nonlinear EEG measures to distinguish externalizing disorders from healthy controls and from anxiety disorders. Additionally, sex assigned at birth improved classification results for both anxiety and externalizing disorders when used with specific ages (3, 5, 7 years for anxiety disorders, 3 and 5 years for externalizing disorders). More detailed and extensive study of covariate influences on electrophysiological biomarkers is needed with larger cohorts. More advanced computational tools that allow joint tensor + matrix factorization may benefit such efforts.

### 4.3. General interpretation of nonlinear measures from EEG signals

Interpretation of nonlinear measures derived from EEG signals in terms of implications for cognitive development and developmental and psychiatric disorders remains challenging. We attempt some general interpretations here. First, it is clear from our results that laminarity, determinism, and entropy vary significantly for age, anxiety disorders, and externalizing disorders. Sample entropy, line length entropy derived from recurrence plots (Lentr), and the vertical entropy (VertEnt) derived from recurrence network analysis each quantify the complexity of the system (i.e., the brain) that produces the EEG time series from which they are computed. Lentr reportedly detects chaos-chaos transitions in the time series, whereas VertEnt detects chaos-periodic transitions. Implications for anxiety disorders are unknown. Although different algorithms are used to compute each, we must presume that they are quantifying a similar dynamical system property, generically called ‘complexity.’ For this reason, it might be expected that all three entropy measures (or none) will be related to phenotypic measures of interest. As mentioned in the brief discussion in Supplemental Table S1, laminarity and determinism are related to fluctuations in the signal between chaotic and laminar or predictable regimes. Theoretical and computational research indicates that the healthy brain operates optimally as a nonlinear dynamical system, poised between totally periodic (as exemplified by a generalized seizure, where all neurons are highly synchronized) and totally random, as exemplified by coma or persistent vegetative states (66). This intermediate region on the dynamical complexity spectrum has been called the edge of chaos critical point (91, 92). Our finding that complexity increases with age, as measured by these measures, is entirely consistent with the edge of chaos theory of optimal brain function.

The relationship between complexity measures and macroscopic conditions such as anxiety or externalizing disorders is more subtle and challenging to explain. It appears that differences in fronto-orbital and central regions may be more important for these disorders than other regions. Laminarity and determinism quantify different aspects of a dynamical system than entropy. Laminarity quantifies the frequency of laminar or non-chaotic states in the time series. The term derives from the early roots of chaotic system theory in characterizations of fluid flow that range from purely stable or laminar to purely random or turbulent. It is related to complexity but characterizes the macroscopic behavior of the system. Similarly, determinism is a quantification of the predictability of the system over short or longer time scales. Our computation of determinism on different frequency scales is also related to predictability of neural electrodynamics over varying time scales. How this relates to anxiety, which might be interpreted as over-sensitivity to internal or external stimuli or a persistent heightened state of arousal, remains to be explored further. Our results suggest that differences in laminarity and determinism are indicative of risk for psychiatric disorders, including anxiety and externalizing disorders. Our finding that different developmental periods were associated with these two disorders suggests that atypical function at different critical developmental windows may be involved.

The entropy measures, which are an indication of the complexity of the EEG signal from which they were derived, reflect the complexity of the neural circuits that directly contribute to the scalp potential measured by the sensor. In general, complexity seems to be associated with neural development. Determinism is related to the predictability of the dynamical system, and laminarity is related to the amount of laminar phases in the system (61). These measure different quantitative characteristics of a dynamical system, but the neural interpretation of these measures is not yet well understood. We speculate that a deeper understanding of the brain as a dynamical system, which is now being explored more fully with reservoir computing (93, 94), may enable better understanding of the meaning of nonlinear analysis of the brain. Accumulation of empirical evidence for neural correlates of specific disorders may help point the way to theoretical explorations.

Future studies are required to validate the precise features and weights needed for a validated digital biomarker for anxiety disorder risk assessment. This will require independent test data to validate the model learned from training data. The probabilistic predictions that are enabled by the supervised tensor factorization algorithm presented here will make such rigorous evaluation possible. It will also be important to continue seeking to understand the behavioral meaning of the nonlinear measures used here. Our approach is based on using EEG time series to reconstruct the essential parameters that describe brain function as a dynamical system that processes information. That is, we are attempting to measure an aspect of neural circuit function at the time of the recording. Whether this is the right set of measurements to predict future neural function, or to detect the neurodevelopmental trajectory, needs further study. Although nonlinear EEG analysis has shown considerable promise for extracting latent neuropsychiatric biomarkers, this does not preclude the (likely) possibility that additional data, such as genetics, other physiological measures, child history of trauma, or task-based analysis of EEG might improve latent biomarkers. Our hope is that the analysis methods presented here will contribute one more tool to the important task of integrating additional data sources into the latent biomarker computation process (56).

### 4.4. Computational methods

In this analysis, we introduced supervised tensor factorization as a digital biomarker scientific discovery tool to examine the utility of EEG data in early life in predicting psychopathology by middle childhood. One of the main advantages of this approach is that multidimensional data, such as the multifrequency nonlinear analysis of EEG signals that was used in this study, is analyzed in a form suitable for the data structure. A great deal more research is needed to extend this method. Necessary extensions include joint factorization of the primary data tensor and the covariate data matrix. Currently, the covariates inform the tensor factorization but are not decomposed and included in the latent variables that are produced. Adding this capability will enable all covariates to be analyzed simultaneously, along with the nonlinear EEG features, to determine the relative contribution of each to the classification or regression task. Unsupervised, coupled tensor-matrix factorization has been accomplished (95), but a supervised version has not. Our results suggest that clinical variables, such as sex assigned at birth and birthweight, may be essential for setting the context for interpretation of functional snapshots of brain activity with EEG. Thus, computational methods that integrate multimodal data may be especially valuable for neurodevelopmental and psychiatric biomarker discovery.

Future research with supervised tensor factorization as a systematic approach to analyzing multiscale nonlinear EEG measures will be needed to determine optimal ways to determine tensor rank and to interpret all of the extracted latent features. It is not yet clear how to select the optimal number of latent factors to extract (i.e., the rank of the tensor), nor is it clear whether only the highest ranked factors are sufficient as biomarkers.

Incorporation of developmental time as an axis when irregular time measurements are to be included is another area of tensor factorization research that will be required for monitoring developmental trajectories by this method. In our analysis, the irregular first year measurement times had to be included as covariates in cases where the infancy data were used. This may explain why inclusion of infancy EEG did not greatly improve the classification results for either disorder. Another weakness in our trajectory modeling is that age is a discrete variable without order in the tensor data structure. A more sophisticated approach that models the time axis as a continuous variable, perhaps even using piecewise linear or polynomial trajectories, might be more appropriate for human neurodevelopmental modeling. However, the current implementation of supervised tensor factorization does not allow this.

Another study limitation is the lower number of EEG measurements available at later ages, particularly at 7 years. Data collection is ongoing, and we thus anticipate having more cases at 7 years (and later) for both anxiety and externalizing disorders, which will enable continued evaluation of classification capabilities using our approach with a larger sample. Importantly, because adolescence is a critical risk period for the development or exacerbation of anxiety, some of the children currently categorized as healthy controls may move to the anxiety disorder group in later development. Longitudinal data collection through adolescence in this sample will allow us to examine the utility of these early neural measures for prediction of anxiety throughout childhood and adolescence. Additionally, because the clinical diagnostic measure inquired about lifetime diagnoses by age 5 years, it is possible that clinical symptoms had emerged prior to or at a similar time as the EEG data collection. Studies that include repeated clinical and contemporaneous EEG measures starting in infancy are needed to determine if the presented approach has value as a predictive indicator of vulnerability to later development of psychopathology and/or as a biomarker reflecting current challenges. Further, applying this method in a sample enriched for risk (e.g., children of parents with a history of a diagnosed anxiety or externalizing disorder) may enhance our ability to identify child risk profiles earlier in development. We hope that this method will move the field forward in identifying specific neural disruptions that may underlie psychopathology in early childhood, enabling the development of more targeted preventative and treatment interventions.

Finally, a deeper probe into correlations among the nonlinear factors used in our calculations may give greater insight into the value and meaning of these factors. As noted previously, the three entropy measures (SE, Lentr and VertEnt) all appeared to be prominent in the latent factors extracted for both disorders. Additionally, DET and LAM appeared to be prominent together. Again, more detailed computational investigation into correlations among these measures may provide additional insights. We note also that many additional nonlinear measures have been utilized by researchers in other domains. For example, at least 20 ‘entropy’ measures can be computed (47).

## 5. Conclusion

The results of this study demonstrate that development may be characterized as a process of increasing complexity that can be measured by nonlinear analysis of EEG signals, which aligns with recent findings that the information processing capacity of dynamical systems increases with system complexity (34, 96). Additional studies to find correlations between complexity measures and cognitive assessments, rather than calendar age, will be an important extension of this study. Although the number of children with anxiety disorders in our study was relatively small, our results indicate that significant information can be extracted from EEG signals to detect or predict risk for anxiety disorders. Externalizing disorders appear to be detectable earlier than anxiety disorders using our approach, although EEG data collected as early as age 3 years helped to distinguish both disorders from healthy controls. Developmental trajectories may be more useful for detecting anxiety and externalizing disorders than any single snapshot in time. We believe this study is the first application of supervised tensor factorization to EEG analysis to find potential biomarkers for anxiety disorders in children. Integration of additional clinical data is possible and required for further development of potential diagnostic, predictive, and monitoring biomarkers for this important childhood disorder.

## Data availability statement

The datasets presented in this article are not readily available because the participant data used for our study was consented specifically for use by researchers affiliated with the Emotion Project at Boston Children’s Hospital. For this reason, the raw EEG data cannot be released publicly. Requests to access the datasets should be directed to michelle.bosquet@childrens.harvard.edu.

## Ethics statement

The studies involving human participants were reviewed and approved by Boston Children’s Hospital Institutional Review Board. Written informed consent to participate in this study was provided by the participants’ legal guardian/next of kin.

## Author contributions

MB and CN conceived of and secured funding for data collection in the Emotion Project, which provided the data for this study. WB and MB designed the computational biomarker approach presented in this study. EL conceived of the supCP algorithm used in this study and wrote the MATLAB code for the algorithm which was used in this study. WB carried out all nonlinear signal processing calculations, performed the tensor factorization, cross validation, and statistical calculations. WB, MB, EL, and CN contributed to discussions of the results and their interpretation. All authors contributed to the article and approved the submitted version.

## Funding

WB, MB, and CN were supported by a grant from National Institute of Mental Health (R01MH078829). WB was supported by a grant from the Koret Foundation through the University of San Francisco. WB and MB were supported by a grant from the Tommy Fuss Center for Neuropsychiatric Disease Research and the Program for Behavioral Science, Department of Psychiatry and Behavioral Sciences, Boston Children’s Hospital. EL was supported by a grant from the National Institute of General Medical Sciences (R01GM130622). The content is solely the responsibility of the authors and does not necessarily represent the official views of any of the funding agencies. Study data were collected and managed using Research Electronic Data Capture (REDCap) tools hosted at Boston Children’s Hospital.

## Conflict of interest

The authors declare that the research was conducted in the absence of any commercial or financial relationships that could be construed as a potential conflict of interest.

## Publisher’s note

All claims expressed in this article are solely those of the authors and do not necessarily represent those of their affiliated organizations, or those of the publisher, the editors and the reviewers. Any product that may be evaluated in this article, or claim that may be made by its manufacturer, is not guaranteed or endorsed by the publisher.

## Supplementary material

The Supplementary material for this article can be found online at: https://www.frontiersin.org/articles/10.3389/fpsyt.2023.1158569/full#supplementary-material

## Footnotes

^1^ https://pypi.org/project/nolds/

^2^ https://pypi.org/project/PyRQA/

^3^ https://github.com/pik-copan/pyunicorn

^4^ https://scikit-learn.org/stable/supervised_learning.html
