# Supplementary material for: A biomarker discovery framework for childhood anxiety
Source: Front Psychiatry. 2023 Jul 17;14:1158569. doi: 10.3389/fpsyt.2023.1158569 (PMC10393248; doi:10.3389/fpsyt.2023.1158569)
Supplement: Supplementary file 1 [file Data_Sheet_1.PDF]

frontiers

Supplementary Material

A Biomarker Discovery Framework for Childhood Anxiety and Externalizing Disorders

William J. Bosl, Michelle Bosquet Enlow*, Eric F. Lock, Charles A. Nelson

*** Correspondence:** Corresponding Author: michelle.bosquet@childrens.harvard.edu

Nonlinear Invariant Variable	Description
**Complexity measures computed using the Python nolds package (https://pypi.org/project/nolds/)**	
Sample Entropy (SampE)	Sample entropy measures the complexity of a time-series. It is precisely the negative natural logarithm of the conditional probability that two sequences similar for *m* points remain similar at the next point, where self-matches are not included in calculating the probability. Its computation is based on approximate entropy, but reduces bias and relative consistency, while being largely independent of signal length. A lower value of sample entropy indicates more self-similarity in a signal and lower complexity.
Correlation Dimension (CD)	Correlation dimension is a measure of the fractal dimension of the phase space of a dynamical system, derived using the Grassberger–Procaccia algorithm.(1) CD has been found to be useful for detecting visual evoked potentials (VEPs) in EEGs, as well as for differentiating sleep stages and detecting Parkinson's Disease and Alzheimer's dementia.(2)
Detrended Fluctuation Analysis (DFA)	DFA is a method for determining the statistical self-affinity of a signal. It is useful for analyzing time series that appear to be long-memory processes (diverging correlation time, e.g. power-law decaying autocorrelation function) or 1/f noise. The obtained exponent is similar to the Hurst exponent, except that DFA may also be applied to signals whose underlying statistics (such as mean and variance) or dynamics are non-stationary (i.e., changing with time).
**Recurrence Plot Measures computed using the Python pyunicorn (http://www.pik-potsdam.de/~donges/pyunicorn/) package**	
Recurrence Rate (RR)	Recurrence rate is the density of recurrence points in a recurrence plot and corresponds with the probability that a specific state will recur. An option used for our calculations is to set a constant RR (0.05 used in this study) rather than a threshold for coloring points to create the recurrence plot.

Determinism (DET)	Determinism is related to dynamical system predictability and is derived from diagonal lines in the recurrence plot as an indicator of deterministic behavior, with a value between 0 and 1. A purely sinusoidal signal will have a value of 1, and a purely stochastic signal will result in a value close to 0.
Laminarity (LAM)	The concept of laminarity is a generalization of laminar flow in fluid dynamics. It is related to the amount of laminar or smooth phases in the system and intermittency or alternation between periodic and chaotic regimes.
Line Length Entropy (Lentr)	Lentr is the Shannon Entropy of the diagonal line lengths in the recurrence plot and reflects the complexity of the deterministic structure in the system. Although not identical, it represents a similar measure to SampEnt, above.
Mean line length (Lmean)	Lmean is the time that two segments of the recurrence plot trajectory are close to each other and can be interpreted as the mean prediction time of the signal, a measure of chaos or divergence from an initial point.
Maximal line length (Lmax)	Lmax is the length of the diagonal lines related to how long segments of the phase space trajectory run parallel, i.e. on the divergence behavior of the trajectories. This concept is related to the maximum Lyapunov exponent, but the two are not exactly equivalent. Lmax describes the divergence of trajectories with small differences in initial states. The higher Lmax, the greater sensitivity to initial conditions, and the less predictable signal behavior.
Trapping time (TT)	Trapping time is an estimate of the time that a system will remain in a given state, such as the length of transition states, as opposed to the time for the transition to take place.
Vertical Entropy (VertEnt)	VertEnt is defined as the entropy of the probability to find a vertical line of exactly length l in the RP. It reflects the complexity of the RP with respect to vertical lines.
Average Vertical Entropy of White lines (AvgWhiteVertEnt)	AvgWhiteVertEnt is another sample entropy value computed from the average white vertical line length distribution, also called mean recurrence time.

Supplementary Table 1. Brief descriptive definitions of the nonlinear measures used in our study, computed on a 30-second EEG interval, are given.
